# Advances in Aquaculture Hatchery Techniques of Sea Urchin *Sphaerechinus granularis* (Lamarck, 1816) (Echinoidea: Toxopneustidae): Broodstock Conditioning and Spawning Induction

**DOI:** 10.3390/life13112233

**Published:** 2023-11-20

**Authors:** Ricardo Luís, Ricardo José, João Castro, Carlos Andrade

**Affiliations:** 1 MARE—Marine and Environmental Sciences Centre, ARNET—Aquatic Research Network, Regional Agency for the Development of Research, Technology and Innovation (ARDITI), 9020-105 Funchal, Portugal; ricardo.jose@oom.arditi.pt (R.J.); carlos.andrade@mare.arditi.pt (C.A.); 2 Regional Agency for the Development of Research, Technology and Innovation (ARDITI), Madeira Tecnopolo, 9020-105 Funchal, Portugal; tiago.castro@madeira.gov.pt

**Keywords:** *Sphaerechinus granularis*, gonadosomatic index, spawning induction, echinoculture

## Abstract

In response to the growing demand for sea urchin gonads (roe or uni) in Asian and European markets and the concerns regarding the overexploitation of wild populations, this preliminary study addresses the need for cost-effective protocols in echinoculture. The primary focus of this research was to evaluate the gonadosomatic index (GI) in captive-conditioned *Sphaerechinus granularis* over a five-month period and compare it with that of their wild-caught conspecifics. Additionally, two different spawning induction methods were assessed: potassium chloride (KCl) injection and agitation. Results indicate that captive-conditioned sea urchins exhibit significantly higher GI values when compared to their wild-caught counterparts. Furthermore, it was observed that the agitation method is equally effective as the KCl injection in triggering a positive response, i.e., gamete ejection, while maintaining lower mortality rates among the subjected *S. granularis*. In conclusion, this preliminary study underscores the pivotal role of broodstock conditioning in supporting the sustainability of sea urchin aquaculture. Moreover, the spawning induction method through agitation emerges as a viable alternative to the traditional intracelomic KCl injection, offering comparable efficacy without compromising the survival of the broodstock. These findings have significant implications for the development of sustainable sea urchin farming practices.

## 1. Introduction

Aquaculture has emerged as a crucial solution to satisfy the ever-growing global demand for all classes of seafood, all the while alleviating the mounting pressure on dwindling wild populations [1,2]. Among the high number of marine species drawing attention in the field of aquaculture, sea urchins have gained recognition due to their culinary and ecological significance [3,4]. One such species is *S. granularis*, commonly referred to as the purple or blunt sea urchin, characterized by its substantial size, up to 150 mm in diameter, twice that of *Paracentrotus lividus*. *S. granularis* displays a typical behavior of utilizing shell fragments, pebbles, and algae as protective coverings, manifesting a cryptic behavior [5]. *S. granularis* are widely distributed across the Mediterranean Sea and the Atlantic coast, ranging from the English Channel to the Gulf of Guinea, including Madeira and other archipelagos of the Macaronesia biogeographic region [6], where they can be used as potential species for diversification in the aquaculture industry. These urchins inhabit the intertidal zones and can be found in depths of up to 130 m [5], boasting a relatively short lifespan of five years but exhibiting a high growth rate compared to their counterparts, *Echinus esculentus* and *P. lividus* [7,8]. Owing to its high market value and the evident signs of overexploitation in urchins’ wild stocks, interest in its aquaculture has been steadily growing [6,9]. While *S. granularis* ranks second in market value after *P. lividus*, these species do not share spawning seasons, which could be attractive for aquaculture by allowing for their culture year-round in dedicated facilities. Additionally, the consistent demand for *S. granularis* gametes and embryos underscores their significance as a model species in ecotoxicology research [10,11,12]. The ecological significance of *S. granularis* cannot be overstated, as these herbivorous sea urchins play a crucial role in maintaining the equilibrium of marine ecosystems. As herbivores, these sea urchins consume macroalgae, contributing significantly to the regulation of algal populations, fostering a healthier marine environment [6]. Considering market dynamics and the challenges associated with the sustainable harvesting of wild populations, diversifying aquaculture species has become imperative. To succeed in aquaculture endeavors and commercial demand, the ability to reproduce offspring in captivity becomes paramount. Equally essential is the comprehension of the factors governing gametogenesis and spawning to enhance egg and larval production [13]. Due to minimal exploitation before 1980, our understanding of *S. granularis* population dynamics, especially their reproductive biology, is still limited. Research employing gonad index methods has shed light on the species’ annual reproductive cycle, unveiling a strong correlation between the onset of spawning and prevailing climatic conditions during the gonadal growth period [6,7]. This pattern encompasses a brief breeding season in May and June, followed by rapid post-spawning recovery and gonad growth throughout July and August [7]. A significant challenge lies in devising efficient husbandry techniques to maximize the gonadosomatic index (GI) [9] and stimulate spawning, all while safeguarding the welfare of the broodstock. Particularly, the successful conditioning of urchin broodstock, accomplished through feeding with maize grains (*Zea mays*), has been achieved for *P. lividus* [13]. Nevertheless, the conventional approach to spawning induction, involving the intracelomic injection of potassium chloride (KCl), while practical, often results in substantial broodstock mortality, thereby delaying sustainability efforts. As a response, alternative induction methods, such as agitation, have demonstrated promise in inducing spawning in other sea urchin species like *P. lividus* [13]. Addressing this challenge stands as an important necessity to meet the increasing demand for sea urchin eggs and larvae without jeopardizing the well-being of the broodstock.

This study aims to address two primary objectives. The first objective revolves around a comparison of the gonadosomatic index (GI) between wild and captive *S. granularis* specimens for a five-month period (August to December 2021), as Lourenço et al., 2022 [6] demonstrated that wild *S. granularis* urchins attain a maximum GI in November (8.03 ± 3.49) with a sharp decline between November and December, when it reaches a minimum (1.69 ± 1.76). The second objective delves into the evaluation of two spawning induction techniques—the KCl injection method and the agitation method—assessing their efficacy in terms of their inducing spawning response and the consequential impact on broodstock survival.

## 2. Materials and Methods

### 2.1. Broodstock Rearing

Wild-caught *S. granularis* specimens with a minimum test size of 50 mm were collected from local wild populations by snorkeling in the subtidal zone at the eastern side of the Madeira Island (Quinta-do-Lorde; 32°74′11.25″ N; 16°70′96.36″ W) in August 2021. The specimens were placed in 25 L containers with natural seawater for their transport (travel time: less than 2 h) to Calheta Mariculture Center (CMC), where they were reared for a five-month period (August to December 2021) in 200 L round tanks with running ambient seawater at a water exchange rate of 45% per hour. Collected individuals were individually sampled for biometric measurements of wet body weight (Pc) (precision scale WTC 600, RADWAG, Radom, Poland), body diameter (Dc) (0.02 mm caliper, DEXTER, China), and body height (Ac) (0.02 mm caliper, DEXTER, China), and randomly placed in the rearing tanks. The animals were then fed with yellow grains of maze *Zea mays*, at 0.7% of the biomass present in the rearing tanks three times a week, and prior to each feeding, the uneaten food and feces were siphoned. The stocking density was (mean ± SD) 8.25 ± 2.94 individuals per square meter, and water quality parameters oxygen (O_2_) (multiparametric meter HandyPolaris, Oxigard^®^, Farum, Denmark), pH (pH Checker, HANNA, Villafranca Padovana, Italy), salinity (refractometer, H_2_Ocean, Essex, UK), and temperature (multiparametric meter HandyPolaris, Oxigard^®^, Farum, Denmark) were registered three times a week. Photoperiod was established at a constant 12-h light/12-h dark for the duration of the assay.

### 2.2. Experiment 1—Gonadosomatic Index (GI)

After a five-month conditioning period, 20 *S. granularis* specimens were randomly selected from the rearing tanks; in addition, 15 *S. granularis* were collected from local wild populations.

The selected urchins for gonadosomatic evaluation were cleaned with filtered and autoclaved seawater (20 µm; 121 °C, 15 min) and then dissected through the oral side, where the gonads were extracted. After removal, wet gonad weight (Pg) was registered (precision scale XT220, Precisa, Switzerland), and GI, in percentage, was calculated with the following equation:

(1) $$GI(\%)=(\frac{Pg}{Pc}\;)\times 100$$

### 2.3. Experiment 2—Spawning Induction Technics

This experiment exclusively employed *S. granularis* specimens reared in captivity. Considering the two spawning induction methods, a total of 70 *S. granularis* urchins were randomly selected from the broodstock in which 30 specimens were used for each spawn induction technique (KCl injection and agitation methods) and 5 specimens as the control group for both induction methods. The control group underwent identical preparation procedures and conditions as the experimental groups, but were not exposed to any spawning induction techniques. All urchins were placed individually with oral side facing down in glass cubes filled with 1.5 L filtered (20 µm) and ultra-violet sterilized (AQUA—UV, De BARY, Deizisau, Germany) seawater for a maximum of 30 min from the beginning of induction procedures.

In KCl spawn induction method, *S. granularis* sea urchins were injected with a volume of 40 µL⋅g^−1^ KCl 0.5 M [13] through the peristome membrane at a 45° angle to urchins’ body periphery to avoid the mouthpiece (Aristotle’s Lantern) and the inadvertent injection of KCl solution in the digestive system. For the agitation spawn induction method, urchins were individually hand-shaken in rotational movements through different rotational vectors, no longer than 60 s. This spawning induction procedure was executed with moderate force to avoid damaging the internal structures of the submitted individuals. Mortality was registered daily for a period of seven days after spawning assays.

When a positive spawning response was observed for each spawning induction method, sex identification was performed by observing the released gametes (oocytes or spermatozoa) under a light microscope (Axioskop 2 plus, Carl Zeiss, Aalen, Germany).

### 2.4. Statistical Analyses

Assays were statistically analyzed separately. In Experiment 1, biometric measurements of wet body weight (Pc), body diameter (Dc), body height (Ac), wet gonad weight (Pg), gonadosomatic index (GI), and gender distribution of *S. granularis* urchins were analyzed for the assessment of the experimental groups’ (five-month captive condition and wild-caught *S. granularis*) biometric uniformity by performing t-Student test (F_t-student=degrees of freedom_ = value; significance level *p*). In Experiment 2, the experimental groups’ (KCl injection, agitation, and control groups) biometric uniformity and seven days of mortality were assessed by performing one-way ANOVA.

Statistical analyses were conducted using IBM SPSS^TM^ Statistics 25 (IBM Corporation, AMONK, New York, NY, USA). All data were tested using the Shapiro–Wilk test for normality and Leven’s test for homogeneity. Parametric data were analyzed using one-way ANOVA (F*_df_* = value; significance level *p*) followed by the post hoc Dunett test. Nonparametric data were analyzed using the Kurskal–Wallis test (H*_df_* = value; significance level *p*) followed by the post hoc Games–Howell test [14]. Results were expressed as mean ± standard deviation (SD), and in all cases, the null hypothesis was rejected when *p* < 0.05 for all statistical analyses.

## 3. Results

### 3.1. Water Quality

Considering the five-month of *S. granularis* conditioning period, the water quality parameters were constant and presented the values (mean ± SD) of dissolved O_2_ = 7.08 ± 0.21 mg⋅L^−1^, pH = 8.18 ± 0.14, salinity = 37.46 ± 0.84, and T = 23.21 ± 1 °C.

### 3.2. Gonadosomatic Index (GI)

In Experiment 1, statistically significant differences were observed in the biometric parameters Pc (F_t-student 33_ = 3.23; *p*-value < 0.05), Dc (F_t-student 33_ = 4.204; *p*-value < 0.05), Ac (F_t-student 33_ = 3.170; *p*-value < 0.05), Pg (F_t-student 33_ = 4.292; *p*-value < 0.05), as well in the GI (F_t-student 33_ = 3.005; *p*-value < 0.05), when compared between five-month captive conditioned *S. granularis* urchins and their wild conspecifics. Statistical analysis revealed that there were no significant differences in the gender category (F_t-student 33_ = 0.574; *p*-value > 0.05) between *S. granularis* experimental groups.

The five-month conditioned *S. granularis* group was characterized by the biometric measurements Pc = 195.55 ± 42.31 g (*n* = 20), Dc = 73.12 ± 5.66 mm (*n* = 20), Ac = 43.97 ± 4.40 mm (*n* = 20), Pg = 10.00 ± 4.01 g (*n* = 20), and the gender distribution was 50% (*n* = 10) M: 50% (*n* = 10) F (males/females). Comparatively, wild-caught *S. granularis* biometric measurements were Pc = 142.56 ± 40.48 g (*n* = 15), Dc = 64.02 ± 7.14 mm (*n* = 15), Ac = 38.97 ± 4.87 mm (*n* = 15), Pg = 4.86 ± 2.67 g (*n* = 15), and the gender distribution was 60% (*n* = 9) M: 40% (*n* = 6) F.

**Figure 1:**
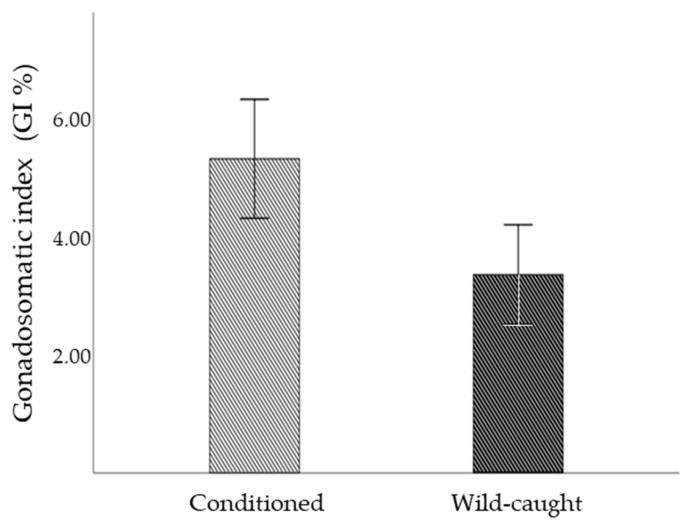
Mean (±SD) gonadosomatic index (%) of 5-month conditioned and wild-caught *Sphaerechinus granularis*.

The observed results showed that conditioning *S. granularis* urchins in captivity for five months prior to the spawning season with maize grains resulted in a significant increase in the gonadosomatic index (GI = 5.30 ± 2.14%) in contrast to wild-caught conspecific sea urchins (GI = 3.34 ± 1.51%) (Figure 1).

### 3.3. Spawning Induction Technics

In Experiment 2, overall biometric measurements were as follows (mean ± SD): Pc = 192.85 ± 52.16 g (*n* = 70), Dc = 72.37 ± 6.37 mm (*n* = 70), and Ac = 44.44 ± 4.41 mm (*n* = 70). Statistical analysis indicated differences in biometric measurements for Pc (F_t-student 3,66_ = 4.576; *p*-value < 0.05), Dc (F_t-student 3,66_ = 4.626; *p*-value < 0.05), Ac (F_t-student 3,66_ = 4.302; *p*-value < 0.05), and mortality (F_t-student 3,66_ = 61.205; *p*-value < 0.05). These differences were found to be statistically significant only for the mortality category of *S. granularis* urchins submitted to KCl injection method (post hoc Dunett; *p* < 0.05).

**Figure 2:**
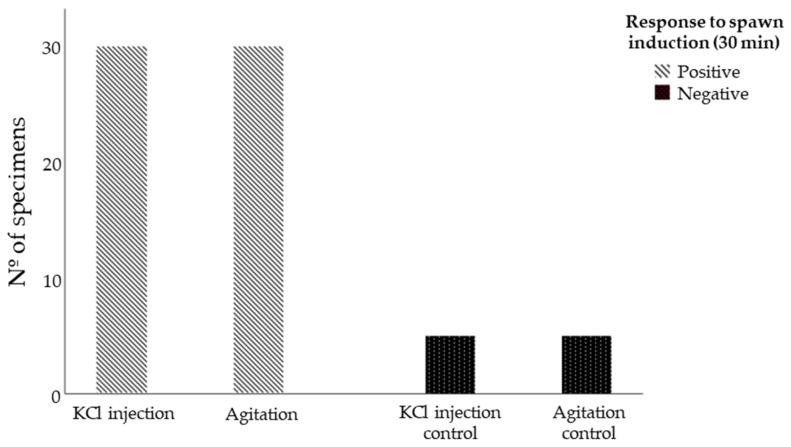
*Sphaerechinus granularis* response to spawn induction methods KCl 0.5 M injection and agitation.

**Figure 3:**
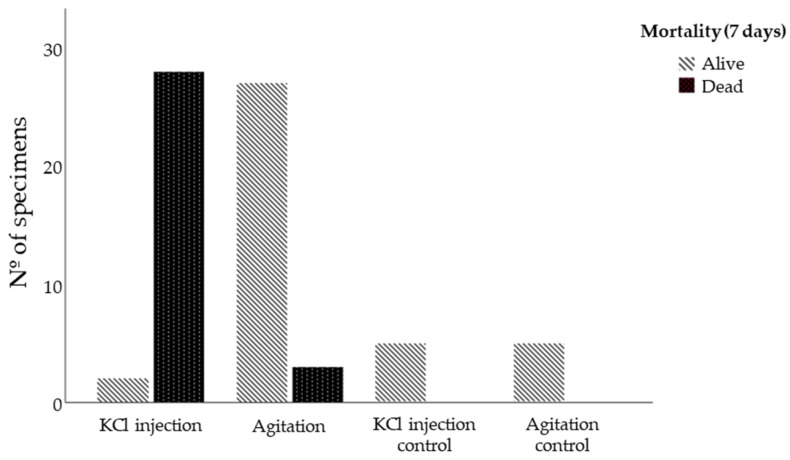
*Sphaerechinus granularis* 7-day mortality when subjected to spawn induction methods KCl 0.5 M injection and agitation.

Results have shown that both induction methods yielded 100% (*n* = 60) positive outcomes, with visible responses occurring within 30 min of the spawning induction procedures (Figure 2). However, the KCl 0.5 M injection resulted in a considerably higher broodstock mortality rate of 93.3% (*n* = 28) within seven days, whereas the agitation method exhibited a significantly lower broodstock mortality rate of 10% (*n* = 3) (Figure 3).

## 4. Discussion

When considering wild urchin populations, wherein their gametogenic cycle is annual and reproduction is generally limited to the spring and summer periods, broodstock captivity is essential to obtain a year-round supply of eggs and larvae. The practice of maintaining sea urchins in captivity has demonstrated a clear capacity to enhance their gonadosomatic index (GI) [9,13,15]. The substantial increase in the gonadosomatic index observed in *S. granularis* over a five-month period in captivity demonstrates the possibility of prolonged captivity as a viable strategy in echinoculture. This finding aligns with the understanding that environmental conditions, dietary factors, and acclimatization all exert influential roles in promoting gonadal development and bolstering the reproductive potential of broodstock [9,13]. This gonadal enhancement is likely attributed to a controlled nutritional intake, the absence of predation, and a reduction in environmental stressors.

Other spawning-inducing techniques have been tested, such as phytoplankton and thermal and saline shocks in *P. lividus*, but have proven to be ineffective [13,15]. When evaluating the efficacy of the two spawning induction methods employed in this study, the success of the agitation technique aligns with the findings of Gago and Luís (2011) in *P. lividus* sea urchins. The agitation method’s ability to induce spawning without significant broodstock loss underscores its potential as a safer alternative to intracelomic KCl injection [16]. Although the latter method offers rapid spawning induction, the substantial mortality rate associated with it represents a significant drawback [13,15] and demonstrates the importance to conduct further investigation to determine the most adequate volume and/or concentration of KCl to use with *S. granularis* urchins. The comparatively lower broodstock mortality rate associated with the agitation method accentuates its superiority in terms of animal welfare and its alignment with sustainable aquaculture practices [13,16]. The present study sheds light on the necessary role of captivity in sea urchin aquaculture and emphasizes the necessity of adopting humane and efficient spawning induction methods that benefit both broodstock and the aquaculture industry.

In summary, this preliminary study underscores the pivotal role of broodstock conditioning during captivity in enhancing the feasibility of sea urchin aquaculture. Conditioning *S. granularis* for a five-month period led to a significant elevation in the gonadosomatic index, which is a key factor for successful reproduction and improved reproductive readiness. Furthermore, the agitation method emerges as a promising and viable alternative to the conventional KCl injection method for spawning induction, demonstrating comparable efficacy while ensuring the survival and welfare of the broodstock. It is essential to recognize that this study represents a preliminary approach, aiming to catalyze further research in this field [16].

These findings carry significant implications for advancing sustainable sea urchin aquaculture practices, providing valuable insights into the best methods for rearing and propagating this species. To further the progress of this field, future research endeavors could delve into refining conditioning protocols, optimizing induction methods and extending the application of these findings to broader aquaculture initiatives [17]. Furthermore, this brief report highlights the need to evaluate the minimum rearing time to obtain a significant increment of *S. granularis* gonads, in addition the evaluation of gamete viability comparing spawning induction methods and other stressors. Ultimately, this preliminary study contributes to the overarching objective of establishing environmentally responsible aquaculture practices that not only meet market demands, but also safeguard the integrity of marine ecosystems.

## Data Availability

The data that support the findings of this study are available from the corresponding author upon reasonable request.

## References

[1] FAO, IFAD, UNICEF, WFP, WHO (2022). The State of Food Security and Nutrition in the World 2022: Repurposing Food and Agricultural Policies to Make Healthy Diets More Affordable.

[2] Lawrence J.M., Zhao C., Chang Y.Q. (2019). Large-scale production of sea urchin (*Strongylocentrotus intermedius*) seed in a hatchery in China. Aquac. Int..

[3] Araújo J., Candeias-Mendes A., Monteiro I., Teixeira D., Soares F., Pousão-Ferreira P. (2020). The use of diatom Skeletonema costatum on aquaculture-produced purple sea urchin (Paracentrotus lividus) larvae and post-larvae diet. Aquac. Res..

[4] Luís R., José R., Castro J., Andrade C. (2023). A Preliminary Assessment of Microalgal Diets for Echinopluteus Larvae Culture of the Sea Urchin *Sphaerechinus granularis* (Lamarck, 1816) Echinoidea: Toxopneustidae). J. Mar. Sci. Eng..

[5] Vafidis D., Antoniadou C., Ioannidi V. (2020). Population density, size structure, and reproductive cycle of the comestible sea urchin *Sphaerechinus granularis* (Echinodermata: Echinoidea) in the Pagasitikos gulf (Aegean Sea). Animals.

[6] Lourenço S., José R., Neves P., Gois A., Cordeiro N., Andrade C., Ribeiro C. (2022). Population Density, Reproduction Cycle and Nutritional Value of *Sphaerechinus granularis* (Echinodermata: Echinoidea) in an Oceanic Insular Ecosystem. Front. Mar. Sci..

[7] Guillou M., Lumingas L.J.L. (1998). The reproductive cycle of the ‘blunt’ sea urchin. Aquac. Int..

[8] Guillou M., Michel C. (1993). Reproduction and growth of *Sphaerechinus granularis* (echinodermata: Echinoidea) in southern Brittany. J. Mar. Biol. Assoc. UK.

[9] Dvoretsky A.G., Dvoretsky V.G. (2020). Aquaculture of green sea urchin in the Barents Sea: A brief review of Russian studies. Rev. Aquac..

[10] García E., Hernández G.C., Clemente S. (2018). Robustness of larval development of intertidal sea urchin species to simulated ocean warming and acidification. Mar. Environ. Res..

[11] Gravina M., Pagano G., Oral R., Guida M., Toscanesi M., Siciliano T., Di Nunzio A., Burić P., Lyons D.M., Thomas P.J. (2018). Heavy rare earth elements affect *Sphaerechinus granularis* sea urchin early life stages by multiple toxicity endpoints. Bull. Environ. Contam. Toxicol..

[12] Pruski A.M., Nahon S., Escande M.L., Charles F. (2009). Ultraviolet radiation induces structural and chromatin damage in Mediterranean sea-urchin spermatozoa. Mutat. Res..

[13] Gago J., Luís O.J. (2011). Comparison of spawning induction techniques on *Paracentrotus lividus* (Echinodermata: Echinoidea) broodstock. Aquac. Int..

[14] Zar J.H. (2010). Biostatistical Analysis.

[15] Brown N., Eddy S. (2015). Echinoderm Aquaculture.

[16] Abou ElMaaty E.E., Hanafy M.H., Yassien M.H., Ghobashy A.F.A., Ahmed M.I., Baeta M. (2023). Induced Spawning and Stocking Density of *Tripneustus gratilla* for aquaculture Proposes in the Red Sea, Egypt. Egypt. J. Aquat. Biol. Fish..

[17] Rahman M.A., Rahman M.H., Asare O.E., Megwalu F.O., Molla M.H.R., Alom M.Z. (2019). Evaluation of growth and production performances of the white sea urchin, *Salmacis sphaeroides* (Linnaeus, 1758) in a captive aqua-rearing system. Aust. J. Sci. Technol..

